# Progress in Psychiatry

**Published:** 1903-11-14

**Authors:** 


### Progress in Psychiatry.

(*Concluded from page* 102.)

**Extraordinary Forms of Multiple Consciousness.**—Dr. Albert Wilson [2] reports a remarkable case of multiple consciousness in the person of a girl who at the age of 12½ years was attacked with influenza and cerebral meningitis lasting six weeks. In the third week she was delirious and maniacal, and showed intense fear of imaginary snakes (illusions or hallucinations). "She was mentally blind in that she could not recognise people, yet a hand or any crease in the counterpane became to her a snake." Choreiform fits and opisthotonos occurred in the fourth week, the attacks being ended by temporary coma. In the fifth week recovery set in and intelligence returned. In the sixth week there developed catalepsy and paraplegia, and quite suddenly one day she had an attack of shaking of the limbs, cried out "it is coming," made some gymnastic movements, and then passed into a new personality in which her manner was childish and her words clipped as in baby talk, and she used wrong words, calling white, black; black, white; red, green. In this state she had some conception of her normal self, whom she called "that person." She also said she was "very cross with that person for going and leaving her." She had frequent cataleptic attacks while in this state, and was noisy and familiar, whereas in her normal state she was a modest and well-behaved child. Other personalities succeeded from time to time in this patient, and as a rule she gave herself a different name in each case. Thus a third one was called by her "Old Nick." This new phenomenon made its appearance on July 24th, 1895, stayed till August 8th, and then disappeared for a year, returning on July 12th, 1896, when it stayed for ten weeks. When in this stage the patient was able to read and write, and enjoyed good health, but displayed a very bad temper. When she returned to a normal state she had no memory of events which had occurred to her in the preceding "Old Nick" state. In the character of a fourth personality she was both deaf and dumb. This deaf mutism recurred five times, having first made its appearance in August 1895. It lasted a few days at a time. Other personalities were of varying character and duration; one was named "good thing" or "good creature" or "pretty dear." This was the most intelligent of the many and learned to speak French. Another was marked by imbecility, blindness, and paraplegia. "The striking feature in this case was that when blind she could draw, while at no other period of her life, either normal or abnormal, had she any ability in drawing." Moral delinquency was exhibited in another stage of personality here. She was violent and cruel, bullying her little sister, and on one occasion she would have forced her into the fire if help had not arrived. When she grew up to be about 16, the normal stage had practically gone for ever. She was sometimes the individual which she called a "thing," but more usually she was another, viz., "good creature." In some of these conditions habits of baby-talk and free forward manners existed, and menstruation, when it did establish itself, made no difference in her mental condition. When about 17 she developed another personality, in which she was self-willed, disobedient to her parents, and subjected to strong attacks of eroticism or inclinations to wantonness. In all, says Dr. Wilson, about a dozen different characters, alternating or occurring at irregular intervals, constituted the total of her psychical life. "This opens," concludes Dr. Wilson, "the very serious question which constantly confronts us—that of responsibility" for acts done in one or other of such states of abnormal personality.

**The Psychopathology of "Seers" and "Mediums."**—Several interesting and recent observations have been recorded on this subject at the Société Médico-Psycologique of Paris [3] by Dr. Gilbert Ballet and others. Gilbert Ballet and Monier Vinard presented the case, which was that of a man who was a professional spiritualistic medium. This person "presented all the characteristics met with in his class, viz., a hyperæsthesia and morbid facility for the development of hallucinations of the senses, especially those of sight and hearing." The variety and mobility of such hallucinations were extreme. At first the man had symptoms indicative of "doubling of personality," which have since become more marked. His present mental status, adds Ballet, recalls that of the visionary Swedenborg, the seer who described the features and forms of life in stars and planets. The patient seems to visit (visually by the medium of a hallucinatory state) the planet Saturn, the appearance of which he describes in detail, including the language and manners of its people. "Saturn is a deserted land," he says, of which "the inhabitants speak a flayed (*écorche*) Greek." "His own body is spirit," he could quit his bed, and, like a smoke or vapour, pass into Saturn. Once there his body becomes grossly material again. His wife's spirit comes to him at times, or makes him write automatically." This patient suffers from ideas of persecution, and is showing signs of commencing mental enfeeblement. The psychological difference between this man and Swedenborg however is that, while the latter was a man of great intellect, the patient is feebleminded. M. Boissier reported an analogous case of a "medium" with mental disequilibrium and hallucinatory tendency. M. Christian [4] stated that he distinguished two categories of mediums, viz., first, those who were rational and fit to live in society at large, and secondly, those who were subject to delusions and who were therefore unsafe to be at large. Such persons attributed their sensory troubles and hallucinations to the influence of spirits sometimes malign, such as the Devil, Astaroth, and Asmodeus. These were likely to develop into cases of demonopathy and "possession." G. Ballet concluded by saying that though various degrees of clairvoyance and mediumship from crystal gazing to automatic spirit-writing were met with, the characteristics common to all seers and mediums was the tendency to the facile dissociation of personality, and in parallelism with this there occurred a proneness to the development of visual hallucinations.

**Recent Views and Observations on General Paralysis.**—Dercum [5] refers to the early diagnosis of general paralysis, and states that in the early stages it is liable to be mistaken for neurasthenia. Yet the symptoms are only superficially similar, those of the neurastheniac being more subjective, while the paretic rarely complains or consults a physician, but is brought by his friends. In neurasthenia the physical appearance is rarely changed, or if so is different to paresis where the facial expression is changed in quality—loss of fine co-ordination and facial expression. The neurastheniac's movements are not altered in precision or co-ordination. The neurastheniac is at his best in the evening, but it is the opposite with the paretic, for then his little delinquencies in the matter of dress, deportment, and speech, become more prominent. The neurastheniac is distinctly nosophobic, and has no essential memory defect, though mental fatigue is readily produced by work. In paresis, in addition to early mental fatigue, there is added loss of ability to remember and to comprehend, impairment of memory is marked, and neglect of attire, of propriety, and of business is manifested, a trait which is foreign to neurasthenia. Paresis may be confused with hypochondria and melancholia. The same factors distinguish it from hypochondria as from neurasthenia. Physical symptoms of paresis appear early. Thus there may be contraction of the visual field without the accompanying retinal hyperæsthesia of neurasthenia. The "paretic manner" so hard to describe, precedes all other physical signs, and consists of slight changes in the subtlety and vivacity of facial expression, speech, and gesture. There is "that something in the eye, the face, the gestures, the words, which reveal that the patient is not in close and accurate touch with his surroundings, with his business affairs, with the fact of his illness, or for that matter with any subject." The significance of a history of syphilitic infection is obvious. It is very difficult to differentiate early paresis from alcoholism, especially where alcoholic excess is one of the first symptoms of paresis. But in incipient insanity resulting from alcoholic indulgence the obtrusion to proprieties and moralities soon cease after discontinuance of the alcohol. Dr. Krauss[6] states that while mental stress, excitement, and excesses lead to neurasthenia in non-syphilitic subjects the syphilitic neurastheniac generally becomes a paretic. It is probable, he thinks, that a state of toxæmia leads to the condition of brain underlying paresis. This toxæmia need not be, and is not, syphilitic, but is probably a toxæmia of bacterial and intestinal origin—a form of auto-intoxication. Dr. L. C. Bruce[7] believes that general paralysis is due to toxæmia brought about by the growth and invasion of bacilli in the gastro-intestinal tract, their point of attack being through the gastro-intestinal mucous membrane. He had previously held that the bacillus coli communis was one of these organisms concerned in the production of paresis but further observations had led him to modify that conclusion and to believe that the bacillus coli infection was a secondary or terminal infection. He had treated eight cases of general paralysis by serum derived from cases of general paralysis in a state of remission. Three of the patients so treated "had apparently made complete recoveries, and were earning their livelihood in the outside world." Of the remaining five patients, one was relieved and four were not improved. Two patients were treated with anti-bacillus coli serum without any beneficial results. Two patients were also treated by means of anti-streptococcus serum; both improved temporarily, but relapsed when treatment was discontinued. One of these two cases, however, made a good recovery when general paralytic serum was employed in treatment. Observations made on the blood of patients suffering with general paralysis showed that in the first stage of the disease the polymorpho-nuclear cells were present in a percentage of 70 or 80. As the disease advanced the polymorpho-nuclear cells decreased in number, until in the third stage of the disease they fell as low as 40 per cent. As the polymorpho-nuclear cells decreased the leucocytis increased. An eosinophilia of the blood was found at some time of the disease in every patient, and varied from 5 to 15 per cent. An eosinophilia was never found in patients who had recovered, or in patients in a state of remission. Dr. Bruce thought that the inducing of a state of hyper-leucocytosis in paretics, by the creation of subcutaneous abscesses, produced great physical and mental improvement, and the disease was temporarily arrested by the high leucocytosis produced. Such abscesses could be produced by the injection of terebinthine. He thought that general paralysis was not a primary cerebral degeneration of the neurons resulting from syphilitic or other poisons, but that it was due to bacterial toxins generated by the growth and invasion of bacteria in the alimentary canal, and that without this bacterial attack there would be no such thing as general paralysis.

[2] Jour. of Ment. Sci., October 1903. [5] Archives de Neurol., June 1903. [4] Ibid. [5] Amer. Jour. of Insan., April 1902. [6] Ibid. [7] Brit. Med. Jour., May 23, 1903, p. 1206.

